# Does prior recall of past week alcohol use affect screening results for at-risk drinking? Findings from a randomized study

**DOI:** 10.1371/journal.pone.0217595

**Published:** 2019-06-04

**Authors:** Andreas Staudt, Jennis Freyer-Adam, Christian Meyer, Gallus Bischof, Ulrich John, Sophie Baumann

**Affiliations:** 1 Institute of Social Medicine and Prevention, University Medicine Greifswald, Greifswald, Germany; 2 German Center for Cardiovascular Research, Partner Site Greifswald, Greifswald, Germany; 3 Institute for Medical Psychology, University Medicine Greifswald, Greifswald, Germany; 4 Department of Psychiatry and Psychotherapy, University Lübeck, Lübeck, Germany; 5 Institute and Policlinic for Occupational and Social Medicine, Medical Faculty, TU Dresden, Dresden, Germany; University of the Witwatersrand, SOUTH AFRICA

## Abstract

Underreporting of alcohol consumption is one of the major challenges in survey research including self-reports. The aim of this study was to test whether underreporting can be reduced by prompting respondents to first reflect on their drinking in the past week and then answer quantity-frequency based screening questions on their typical alcohol use. Data come from 2,379 adults (54% female; mean age = 31.8 years, *SD* = 11.4 years) consecutively recruited at a local registration office in northeastern Germany. Participants responded to an electronic, self-administered questionnaire on different health behaviors. They were randomized to receiving the Alcohol Use Disorders Identification Test—Consumption (AUDIT-C) either before or after the assessment of past week timeline follow-back questions. Logistic regression models were calculated predicting positive screening results for at-risk drinking. Potential interaction effects with gender, age and educational background were explored. Results show that the assessment of past week alcohol consumption prior to the assessment of the AUDIT-C reduced the odds of obtaining positive screening results (*OR* = 0.83; *95% CI* = 0.70–0.99). There were no interaction effects with gender, age and educational background. As a secondary finding, participants reported consistently lower alcohol consumption in the alcohol measure that was administered later in the questionnaire. Preceding questions about alcohol consumption in the past week reduced the probability of positive screening results for at-risk drinking. Our findings suggest that prompting people to recall past week alcohol use prior to screening may not be a solution to reduce underreporting.

## Introduction

Misreporting of alcohol consumption is a problem in population surveys: people struggle in reporting their actual alcohol consumption accurately [1]. Misreporting on self-report measures may compromise the detection of individuals with hazardous alcohol consumption. Furthermore, misreporting attenuates the quality of evidence that is used to estimate the risk for alcohol-related problems and to derive low-risk drinking guidelines [2, 3]. While both over- and underreporting of alcohol consumption have been observed, the gap between self-reported alcohol consumption and estimates derived from alcohol sales data indicates that underreporting is the more severe problem [4]. Underreporting has been found to vary by gender and age [5, 6], drinking pattern [4, 7] as well as by features of the research instrument [8]. If quantity-frequency items and retrospective diary methods were compared within the same sample, the latter would yield substantively higher estimates of average alcohol consumption [9–11].

The magnitude of underreporting may partially be explained by recall errors [9, 12]. For instance, one of the most widely used screening instruments for hazardous alcohol use, the Alcohol Use Disorders Identification Test–Consumption (AUDIT-C) [13], assesses respondents’ self-reported typical frequency and quantity of alcohol consumption [14]. In order to provide valid answers, respondents are expected to consider and reflect on past drinking episodes that have to be condensed to a global appraisal of typical drinking *frequency*. It is questionable whether respondents perform such a complex cognitive operation. It seems unlikely that they have detailed episodic representations available, unless the behavior is rare and of considerable importance [8]. Concerning frequent behavior, it seems more likely that respondents roughly estimate their drinking by drawing upon schemas stored in memory [15] rather than counting individual episodes. According to cognitive psychology, schemas are structured units of knowledge that contain easily accessible information about subjects, events or the self [16]. These estimates may be imprecise and open to bias [12]. Imprecision may be an issue for the assessment of typical drinking *quantity*, too. Respondents are expected to provide information on average quantity of alcohol consumed. However, there may be large variations in quantities within one person [17]. As a consequence, the purpose of the quantity-frequency approach may be undermined by human’s memory constraints. The respondents’ way of obtaining their respective answers may be based on schemas rather than on accurate episodic memories as intended by the measure.

Besides recall deficits, underreporting may also be explained by general tendencies towards socially desirable responding [18] or by bias resulting from specific social context factors [19]. Respondents may be influenced by social norms that are salient in a given situation [20], the interview situation [21], the interview setting [22] or the way the respondents perceive the intention of the interviewer [12] as well as their anonymity [23].

Nevertheless, quantity-frequency based measures assessing typical behavior such as the AUDIT-C [24] are recommended and frequently used. Retrospective measures with short reference periods may be less prone to recall bias [19], but may disregard seasonal [25], event-specific [26] or even random variability in alcohol consumption [12, 27]. Prospective measures may be expected to yield most valid information [1] but seem impractical for routine care and brief interventions.

As outlined above, respondents are likely to roughly estimate their typical drinking frequencies and quantities based on available schemas [8]. Schemas are applied and updated in comparison with new experiences [16]. Given the assumption that schemas contain an underestimate of the respondents’ true alcohol consumption, respondents might be prompted to correct their schema upwards when confronted with memories of more intense drinking. Recent drinking can be expected to be higher than respondents’ reported average consumption [9–11, 28, 29]. Regarding the assessment of alcohol use, the content of preceding questions can shape response behavior in subsequent ones [12, 30, 31]. Therefore, we hypothesized that preceding questions about alcohol consumption in the past week may reduce underreporting in a subsequent quantity-frequency based screening measure as respondents may become more aware of the discrepancy between their most recent consumption and their estimated average.

The aim of the present study was to test (i) whether underreporting of alcohol consumption may be reduced by prompting respondents to reflect on their drinking in the past week prior to the assessment of frequency and quantity of drinking, and (ii) whether this effect is moderated by socio-demographic variables, namely gender, age and school education.

## Materials and methods

Data were collected as part of the randomized controlled trial “Testing a proactive expert system intervention to prevent and to quit at-risk alcohol use” (PRINT, German Clinical Trials Register: DRKS00014274, date of registration: 2018/03/12) described in more detail elsewhere [32]. The ethics committee of the University Medicine Greifswald approved the study (protocol number BB 147/15).

### Participant recruitment and study procedure

Participants were recruited from the general population. In April and May 2018, all clients aged 18 to 64 years who appeared in the waiting area of the registration office in Greifswald, Mecklenburg-Western Pomerania, Germany, were approached by study assistants. The registration office is the public authority for registration, passport and vehicle admission issues in Germany. Clients were asked to fill in a questionnaire on health behaviors. Clients cognitively or physically incapable, clients with insufficient language or reading skills, clients having already been approached during an earlier visit, escorting persons and clients employed at our research institute were excluded.

Clients received oral and written information about study purpose, data handling and anonymity. Those who agreed received a tablet computer, and were briefly instructed into the handling of the tablet-based, self-administered questionnaire. All participants recorded their informed consent electronically on the tablet computer prior to participation. Written informed consent and personal data was obtained from those participants who were eligible to participate and who agreed to participate in the PRINT trial. This procedure was approved by the ethics committee of the University Medicine Greifswald (protocol number BB 147/15).

Completing the questionnaire took 5 to 10 minutes. Participants who reported alcohol use in the past 12 months (“Did you drink alcohol in the past 12 months?”) received a detailed alcohol assessment (i.e. screening for at-risk alcohol consumption, past week drinking, and motivational constructs related to alcohol consumption) and comprised the final sample analyzed in this study.

### Experimental conditions

Participants were assigned to study conditions using a random generator implemented in the tablet computers. This randomization affected all participants who received the detailed alcohol assessment. One condition was first asked to recall past week alcohol use before responding to the alcohol screening measure (*Screening with prior past week recall*). The other condition was asked to respond to the alcohol screening measure first (*Screening without prior past week recall*). In this condition, past week alcohol use was also assessed, but after the screening. Participants were unaware that this randomization took place.

### Measures

#### Alcohol use measures

The detailed alcohol assessment included (i) the Alcohol Use Disorders Identification Test (AUDIT) [33] as screening measure, and (ii) timeline follow-back questions (TLFB) [34] to ask for alcohol use in the past week.

Concerning the screening measure, the AUDIT-C [13] was used to screen for at-risk alcohol consumption. The AUDIT-C has been validated in general population samples [14, 35] and showed very good sensitivity in detecting at-risk drinking. Regarding specificity, results were mixed [14, 35], depending on the cut-off value used. We used cut-off scores of ≥ 4 for women and ≥ 5 for men [36]. The third item (“How often do you have 4 [for women] / 5 [for men] or more alcoholic drinks on one occasion?”) was adapted to gender-specific limits of current low-risk drinking guidelines [24, 37]. Beyond that, answers on the first two items (“How often do you have a drink containing alcohol?” and “How many drinks do you have on a typical day when you are drinking?”) were computed into an index for average weekly alcohol consumption by assuming the median of each response option.

Concerning the past week recall, TLFB questions [34] ask participants to indicate the number of alcoholic drinks they had on each of the seven days prior to the assessment. For both the AUDIT and TLFB items, participants were informed about the concept of standard drinks with a note displayed on the tablet screen that included exemplary beverages. A drink was defined as 0.25–0.3l beer, 0.1–0.15l wine or sparkling wine or 4cl spirits.

#### Covariates

Gender, age, educational background, and relationship status were assessed. Educational background included 9 years or less, 10 to 11, and 12 or more years of school education. Relationship status was coded one when currently married or living in a partnership, and zero when not. Furthermore, smoking status (never, former, current smoker) was assessed.

### Statistical analysis

Differences between experimental conditions were tested using two-sided t-tests, χ^2^-tests or Mann-Whitney-U-tests with *p* < .05 as significance level. In order to test whether prior past week recall of alcohol use affects screening results, logistic regression models were calculated. Data were analyzed in three steps. First, at-risk alcohol consumption according to the AUDIT-C score (0 = low-risk alcohol use, 1 = at-risk alcohol use) was regressed on experimental condition (0 = screening without prior past week recall, 1 = screening with prior past week recall) (unadjusted model). Second, gender, age, educational background, relationship status and smoking status were added as covariates (adjusted model). Linearity of age as continuous predictor and log odds was tested with graphical analysis using the LOWESS technique and the Box Tidwell Transformation Test [38]. As the assumption of linearity was violated, we collapsed age into a categorical variable with three groups (18–29, 30–45 and 46–64 year-olds). In a third step, we explored whether gender, age or educational background moderated the effect of experimental condition on screening by adding respective interaction terms into our regression model. Results of logistic regression models were given as odds ratios (OR), 95% confidence intervals (CI) and exact *p*-values. All statistical analyses were carried out using Stata 14 [39]. As the tablet computers did not allow for skipping items without providing an answer to the respective question and we had no data loss due to technical reasons, there was no missing data.

## Results

### Sample characteristics

**Fig 1:**
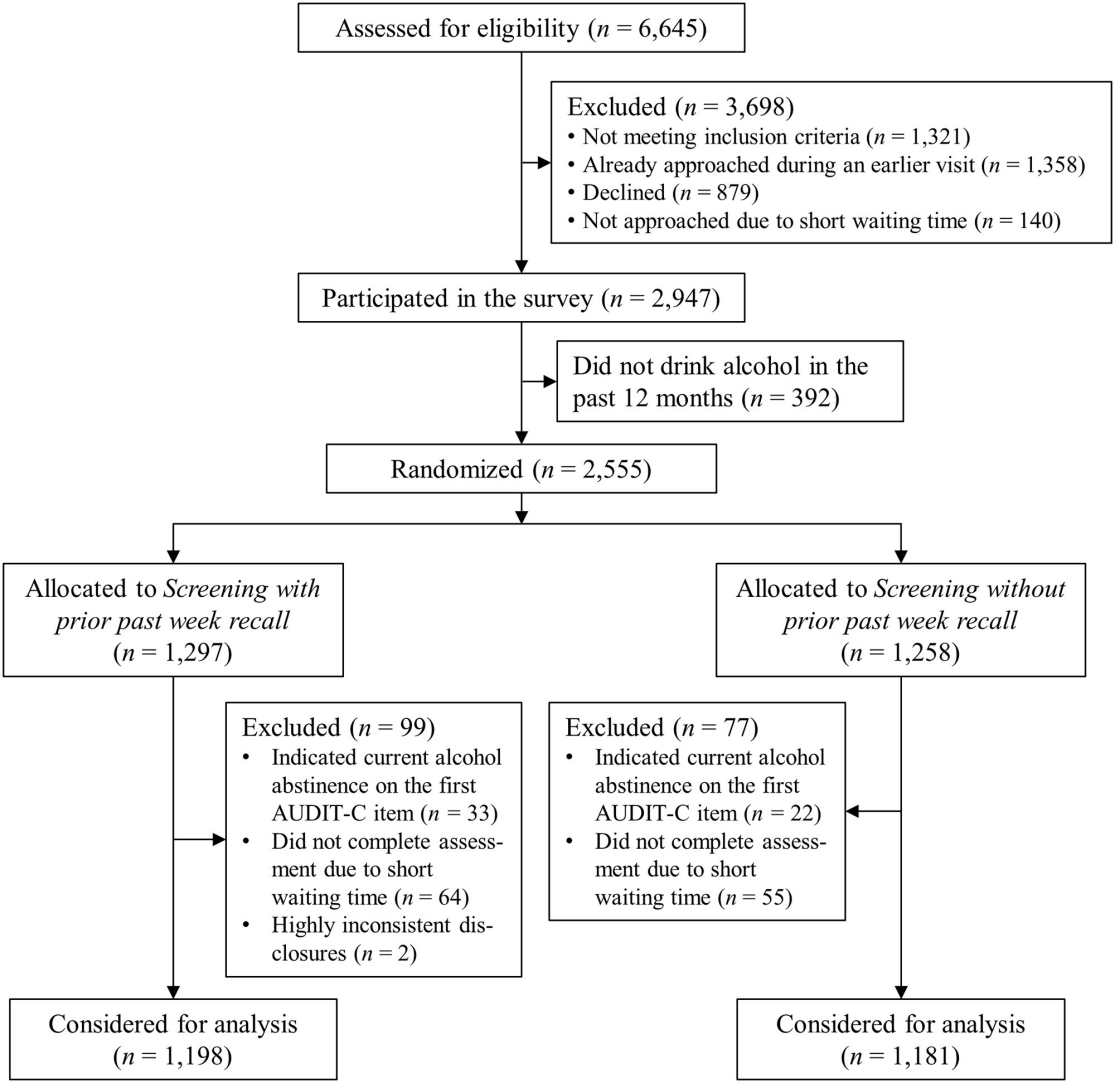
Flow of participants.

Overall, 6,645 persons appeared in the waiting area during our recruitment period. Among them, 3,966 were eligible for our survey. Of all eligible clients, 2,947 (74.3%) participated in the survey. Of these, 392 reported no alcohol use in the past 12 months and did not receive the detailed alcohol assessment. Randomization took place for 2,555 participants (Fig 1), of whom 1,297 were assigned to *Screening with prior past week recall* and 1,258 to *Screening without prior past week recall*. Among the 2,555 randomized participants, 119 did not complete the assessment due to insufficient waiting time in the registration office. Further 55 participants, who received the detailed alcohol assessment, indicated current alcohol abstinence on the first AUDIT-C item. These participants were excluded from analysis. Two persons with highly inconsistent disclosures (daily drinking of more than 800 grams of alcohol on the past week items and drinking *less than once per month* on the first AUDIT-C item) were also excluded. The final sample to be used for analysis encompassed 2,379 participants.

The final sample (*N* = 2,379; 54.3% female) had a mean age of 31.8 years (*SD* = 11.4). Sixty-two percent (*n* = 1,482) had 12 or more, 31.2% (*n* = 742) 10 to 11 and 6.5% (*n* = 155) had 9 or less years of schooling. Current smoking was reported by 33.5% (*n* = 798). There were no statistically significant differences between the two experimental conditions regarding socio-demographic characteristics or smoking (Table 1).

**Table 1:** Sample characteristics and alcohol measures.

		Experimental condition: Screening…		
	Overall	…with prior past week recall	…without prior past week recall	
**Socio-demographics**	*n* = 2,379	*n* = 1,198	*n* = 1,181	*p*
Women, *n* (%)	1,292 (54.3%)	632 (52.8%)	660 (55.9%)	.125^b^
Age in years, *M* (*SD*)	31.8 (11.4)	31.7 (11.2)	31.8 (11.6)	.897^a^
Age groups, *n* (%)				
18–29 year-olds	1,290 (54.2%)	651 (54.3%)	639 (54.1%)	
30–45 year-olds	732 (30.8%)	376 (31.4%)	356 (30.1%)	.558^b^
46–64 year-olds	357 (15.0%)	171 (14.3%)	186 (15.8%)	
Educational background, *n* (%)				
≤ 9 years	155 (6.5%)	80 (6.7%)	75 (6.4%)	
10–11 years	742 (31.2%)	399 (33.3%)	343 (29.0%)	.062^b^
≥ 12 years	1,482 (62.3%)	719 (60.0%)	763 (64.6%)	
In a relationship, *n* (%)	1,542 (64.8%)	777 (64.9%)	765 (64.8%)	.966^b^
Smoking status, *n* (%)				
Nonsmokers	1,197 (50.3%)	592 (49.4%)	605 (51.2%)	
Former smokers	384 (16.1%)	199 (16.6%)	185 (15.7%)	.653^b^
Current smokers	798 (33.5%)	407 (34.0%)	391 (33.1%)	
**Alcohol consumption**				
At-risk alcohol consumption according to AUDIT-C^1^, *n* (%)	846 (35.6%)	402 (33.6%)	444 (37.6%)	.040^b^
Average number of drinks per week (AUDIT-C), *M* (*SD*)	2.9 (4.3)	2.9 (3.9)	3.0 (4.7)	.692^c^
Number of alcoholic drinks in the past week (TLFB), *M* (*SD*)	5.0 (7.2)	5.6 (7.5)	4.4 (6.9)	< .001^c^
AUDIT-C score, *M* (*SD*)	3.6 (1.8)	3.5 (1.8)	3.6 (1.8)	.420^c^

AUDIT-C = Alcohol Use Disorders Identification Test–Consumption. ^1^At-risk alcohol consumption cut-off values according to AUDIT-C: sum score of 4 or more for females and 5 or more for males. TLFB = Timeline follow-back items referring to the last 7 days. *p*-values from two-sided t-tests^a^ (*df* = 1,377), χ^2^-tests^b^ and Mann-Whitney-U-tests^c^.

### Alcohol measures across conditions

According to the first two AUDIT-C items, the average weekly alcohol consumption in our sample was 2.9 alcoholic standard drinks (*SD* = 4.3). In the week prior to their respective assessment, participants drank on average 5.0 alcoholic standard drinks (*SD* = 7.2). There were no differences between experimental conditions concerning average weekly consumption (*p* = .692) and AUDIT-C score (*p* = .420). The condition *Screening with prior past week recall* reported a significantly higher number of alcoholic beverages in the TLFB items (*p* < .001), and revealed a lower percentage of positive screenings for at-risk alcohol consumption (*p* = .040) than the condition *Screening without prior past week recall*.

### Logistic regression models

The unadjusted model revealed that experimental condition significantly predicted screening result (*OR* = 0.84; *95% CI*: 0.71–0.99). This finding remained significant in the adjusted model (*OR* = 0.83; *95% CI*: 0.70–0.99; Table 2). The three subsequently conducted logistic regression models revealed that neither gender (*p* = .977), nor age (*p* = .603 and *p* = .081), nor educational background (*p* = .613 and *p* = .796) moderated the effect of experimental condition on screening result.

**Table 2:** Logistic regression models predicting positive AUDIT-C screening.

	*Odds Ratio*	*95% CI*	*p*-value
Unadjusted model			
Experimental condition *Reference*: Screening without prior past week recall	0.84	0.71; 0.99	.040
Adjusted model			
Experimental condition *Reference*: Screening without prior past week recall	0.83	0.70; 0.99	.041
Gender *Reference*: Males	1.25	1.04; 1.49	.016
Age groups *Reference*: 18–29 year-olds			
30–45 year-olds	0.58	0.47; 0.72	< .001
46–64 year-olds	0.59	0.44; 0.78	< .001
Educational background *Reference*: ≤ 9 years			
10–11 years	1.78	1.19; 2.67	.005
≥ 12 years	2.92	1.97; 4.33	< .001
Relationship status *Reference*: Not in a relationship	0.78	0.65; 0.95	.011
Smoking status *Reference*: Nonsmokers			
Former smokers	2.11	1.62; 2.74	< .001
Current smokers	3.49	2.82; 4.32	< .001

*N* = 2,379 for both models.

## Discussion

Our study revealed two main findings. First, respondents who were asked to recall their past week alcohol use before responding to the AUDIT-C had reduced odds of receiving a positive screening result. Furthermore, in both conditions the alcohol measure that was assessed first revealed higher values in comparison to the other condition, in which the respective measure was assessed second.

Our study revealed that underreporting of alcohol consumption in screening measures may not be reduced by preceding questions about alcohol consumption in the past week. In accordance with previous studies [9–11, 28, 29], we found that respondents reported higher consumption for a short recall period (the past week) compared to a more general assessment of typical alcohol consumption as assessed by the AUDIT-C. This finding supports the notion that our respondents underreported typical frequency and quantity of their alcohol consumption in the AUDIT-C. However, our hypothesis that respondents would report higher alcohol use in quantity-frequency based AUDIT-C screening following their recall of alcohol use for each day in the past week was not supported. The opposite was found. Although the actual underlying cognitive processes remain unclear, fewer positive screening results for at-risk alcohol consumption were obtained when respondents were asked to recall past week alcohol use first, even after controlling for gender, age, educational background, relationship status and smoking status. Thus, it has to be acknowledged that we were not able to reduce underreporting in screening for at-risk drinking merely by prompting respondents to recall past week alcohol use before its assessment. However, two alternative explanations are also likely for our unexpected results.

Firstly, respondents might have become aware of our particular interest in their drinking behavior when they were faced with the second alcohol measure (either AUDIT or past week alcohol use). The second alcohol measure may have initiated tendencies towards social desirable answers leading to underreporting particularly in the second measure. Misreporting due to social desirability is influenced by the perceived context of the assessment [40]. As the screening was introduced as a survey on different health behaviors, the administration of multiple alcohol measures may have led our respondents to suspect that we have a particular interest in their alcohol consumption. For those for whom the AUDIT was the second measure, responses may have been biased by social desirability in order to downplay past week alcohol use. Our assumption that the second alcohol measure initiated tendencies towards social desirable answers is also supported by the finding that significantly fewer alcoholic drinks were reported in the past week when the past week items were presented after the AUDIT. This points towards a potential source of error in surveys; more alcohol measures within a questionnaire might elicit underreporting and lead to increased bias of participants’ disclosures in later alcohol measures.

Secondly, irregular heavy episodic drinking may have caused these results. Apart from typical quantity and frequency of alcohol consumption, heavy episodic drinking is an integral part of hazardous drinking [37]. Underreporting has been shown to be particularly marked among people who engage in heavy episodic drinking infrequently [5]. Infrequent heavy episodic drinkers represent the majority of our sample according to the third AUDIT-C item: 68% indicated to engage in heavy episodic drinking less than once per month. The past week as a time frame may have been too short to include heavy drinking episodes for these people. Thus, administering past week items prior to the AUDIT may have had the undesired effect of prompting our respondents to underestimate their heavy episodic drinking frequency, as the recent recall period most likely did not include such an episode.

Two limitations have to be addressed. First, although the proportion of individuals who participated in our study among the eligible (74%) is acceptable, selection bias is likely. For instance, our sample is not representative of the German general population in terms of educational background: 62% of our sample experienced 12 or more years of schooling. Based on census data, the proportion of 15- to 64-year-olds with 12 or more years of schooling among the general population is only 31% [41]. The town in which the study was conducted is characterized by a large proportion of university students, i.e. 10,247 university students [42] and 58,886 inhabitants [43]. Second, our study lacked external validation data, i.e. we did not have information on the actual amount of alcohol consumed to validate self-reports. This study was based on the common assumption in alcohol research that higher reported alcohol consumption is closer to the true amount of alcohol consumed [5, 29]. However, whether this is true remains afflicted with uncertainty. The gap between self-reported alcohol consumption and estimates derived from alcohol sales data [4] may not only be attributable to underreporting but also to other factors such as systematic sampling errors in population surveys.

Our findings suggest that prompting people to recall past week alcohol use prior to screening may not be a solution to reduce underreporting. Our findings even suggest that the opposite may be true. Putting recent drinking episodes into the focus of attention may not improve the recall of episodic memories required for screening purposes but rather trigger social desirable answers or neglect of more intensive drinking episodes. Furthermore, assessing more than one alcohol measure may amplify underreporting in any subsequent alcohol measure. Identifying the determinants of underreporting in alcohol surveys and finding potential remedies, for instance by means of audio-guided computer-assisted self-interviews [44] or alternative question formats such as within-location beverage-specific questions [5], remains a worthwhile endeavor in order to prevent false negative screening results and missing people in need for intervention.

## Supporting information

S1 Dataset Study data. (DTA)

## References

[1] Gmel G, Rehm J. Measuring alcohol consumption. Contemp Drug Probl. 2004;31(3):467–540.

[2] Stockwell T, Butt P, Beirness D, Gliksman L, Paradis C. The basis for Canada’s new low-risk drinking guidelines: a relative risk approach to estimating hazardous levels and patterns of alcohol use. Drug Alcohol Rev. 2012;31(2):126–34. 10.1111/j.1465-3362.2011.00342.x 21954872

[3] Stockwell T, Room R. Constructing and responding to low-risk drinking guidelines: conceptualisation, evidence and reception. Drug Alcohol Rev. 2012;31:121–5. 10.1111/j.1465-3362.2011.00416.x 22385130

[4] Stockwell T, Zhao J, Greenfield T, Li J, Livingston M, Meng Y. Estimating under- and over-reporting of drinking in national surveys of alcohol consumption: identification of consistent biases across four English-speaking countries. Addiction. 2016;111:1203–13. 10.1111/add.13373 26948693 PMC4899158

[5] Livingston M, Callinan S. Underreporting in Alcohol Surveys: Whose Drinking Is Underestimated? J Stud Alcohol Drugs. 2015;76(1):158–64. 10.15288/jsad.2015.76.158 25486405

[6] Stockwell T, Zhao J, Macdonald S. Who under-reports their alcohol consumption in telephone surveys and by how much? An application of the ‘yesterday method’ in a national Canadian substance use survey. Addiction. 2014;109(10):1657–66. 10.1111/add.12609 24825591

[7] Boniface S, Kneale J, Shelton N. Drinking pattern is more strongly associated with under-reporting of alcohol consumption than socio-demographic factors: evidence from a mixed-methods study. BMC Public Health. 2014;14 10.1186/1471-2458-14-1297 25519144 PMC4320509

[8] Schwarz N. Self-Reports: How the Questions Shape the Answers. American Psychologist. 1999;54(2):93–105.

[9] Heeb JL, Gmel G. Measuring alcohol consumption: a comparison of graduated frequency, quantity frequency, and weekly recall diary methods in a general population survey. Addict Behav. 2005;30(3):403–13. 10.1016/j.addbeh.2004.04.022 15718058

[10] Shakeshaft AP, Bowman JA, Sanson-Fisher RW. A comparison of two retrospective measures of weekly alcohol consumption: diary and quantity/frequency index. Alcohol Alcohol. 1999;34(4):636–45. 10.1093/alcalc/34.4.636 10456593

[11] Utpala-Kumar R, Deane FP. Rates of alcohol consumption and risk status among Australian university students vary by assessment questions. Drug Alcohol Rev. 2010;29(1):28–34. 10.1111/j.1465-3362.2009.00082.x 20078679

[12] Greenfield TK, Kerr WC. Alcohol measurement methodology in epidemiology: recent advances and opportunities. Addiction. 2008;103(7):1082–99. 10.1111/j.1360-0443.2008.02197.x 18422826 PMC2782942

[13] Bush K, Kivlahan DR, McDonell MB, Fihn SD, Bradley KA. The AUDIT alcohol consumption questions (AUDIT-C): An effective brief screening test for problem drinking. Arch Intern Med. 1998;158:1789–95. 9738608 10.1001/archinte.158.16.1789

[14] Dawson DA, Grant BF, Stinson FS, Zhou Y. Effectiveness of the Derived Alcohol Use Disorders Identification Test (AUDIT-C) in Screening for Alcohol Use Disorders and Risk Drinking in the US General Population. Alcohol Clin Exp Res. 2005;29(5):844–54. 10.1097/01.alc.0000164374.32229.a2 15897730

[15] Bradburn N, Sudman S, Wansink B. Asking Questions. San Francisco: Jossey-Bass; 2004.

[16] Eysenck MW, Keane MT. Cognitive psychology: a student’s handbook. 7th ed London, New York: Psychology Press, Taylor & Francis Group; 2015.

[17] Dawson DA. Methodologial Issues in Measuring Alcohol Use. Alcohol Res Health. 2003;27(1):18–29. 15301397 PMC6676704

[18] Davis CG, Thake J, Vilhena N. Social desirability biases in self-reported alcohol consumption and harms. Addict Behav. 2010;35:302–11. 10.1016/j.addbeh.2009.11.001 19932936

[19] Del Boca FK, Darkes J. The validity of self-reports of alcohol consumption: state of the science and challenges for research. Addiction. 2003;98:1–12. 10.1046/j.1359-6357.2003.00586.x 14984237

[20] Chung A, Rimal RN. Social norms: a review. Review of Communication Research. 2016;4:1–29. 10.12840/issn.2255-4165.2016.04.01.008

[21] Fadnes LT, Taube A, Tylleskär T. How to identify information bias due to self-reporting in epidemiological research. Int J Epidemiol. 2009;7(2).

[22] Bradley KA, Lapham GT, Hawkins EJ, Achtmeyer CE, Williams EC, Thomas RM, et al Quality concerns with routine alcohol screening in VA clinical settings. J Gen Intern Med. 2011;26(3):299–306. 10.1007/s11606-010-1509-4 20859699 PMC3043188

[23] Beatty JR, Chase SK, Ondersma SJ. A randomized study of the effect of anonymity, quasi-anonymity, and Certificates of Confidentiality on postpartum women’s disclosure of sensitive information. Drug Alcohol Depend. 2014;134:280–4. 10.1016/j.drugalcdep.2013.10.016 24246900

[24] Higgins-Biddle JC, Babor TF. A review of the Alcohol Use Disorders Identification Test (AUDIT), AUDIT-C, and USAUDIT for screening in the United States: Past issues and future directions. Am J Drug Alcohol Abuse. 2018;44(6):578–86. 10.1080/00952990.2018.1456545 29723083 PMC6217805

[25] Knudsen AK, Skogen JC. Monthly variations in self-report of time-specified and typical alcohol use: the Nord-Trondelag Health Study (HUNT3). BMC Public Health. 2015;15:1–11. 25884177 10.1186/s12889-015-1533-8 PMC4360933

[26] Kushnir V, Cunningham JA. Event-Specific Drinking in the General Population. J Stud Alcohol Drugs. 2014;75:968–72. 10.15288/jsad.2014.75.968 25343654 PMC4211338

[27] Staudt A, Freyer-Adam J, Meyer C, John U, Baumann S. Short-term stability of different drinking patterns over the course of four weeks among adults. A latent transition analysis. Drug Alcohol Depend. 2018;191:181–6. 10.1016/j.drugalcdep.2018.06.031 30125760

[28] Townshend JM, Duka T. Patterns of alcohol drinking in a population of young social drinkers: a comparison of questionnaire and diary measures. Alcohol Alcohol. 2002;37(2):187–92. 10.1093/alcalc/37.2.187 11912076

[29] Stockwell T, Donath S, Cooper-Stanbury M, Chikritzhs T, Catalano P, Mateo C. Under-reporting of alcohol consumption in household surveys: a comparison of quantity-frequency, graduated-frequency and recent recall. Addiction. 2004;99(8):1024–33. 10.1111/j.1360-0443.2004.00815.x 15265099

[30] Bischof G, Reinhardt S, Groethues J, Dybek I, Meyer C, Hapke U, et al Effects of item sequence on the performance of the AUDIT in general practices. Drug Alcohol Depend. 2005;79:373–7. 10.1016/j.drugalcdep.2005.03.002 16102379

[31] Harford TC. The effects of order of questions on reported alcohol consumption. Addiction. 1994;89:421–4. 8025495 10.1111/j.1360-0443.1994.tb00916.x

[32] Baumann S, Staudt A, Freyer-Adam J, John U. Proactive expert system intervention to prevent or quit at-risk alcohol use (PRINT): study protocol of a randomized controlled trial. BMC Public Health. 2018;18(1):851 10.1186/s12889-018-5774-1 29986695 PMC6038316

[33] Saunders JB, Aasland OG, Babor TF, De La Fuente J, R., Grant M. Development of the Alcohol Use Disorders Identification Test (AUDIT): WHO Collaborative Project on Early Detection of Persons with Harmful Alcohol Consumption. Addiction. 1993;88:791–804. 8329970 10.1111/j.1360-0443.1993.tb02093.x

[34] Sobell LC, Sobell M. Timeline follow-back: A technique for assessing self-reported alcohol consumption In: Litten RZ, Allen JP, editors. Measuring Alcohol Consumption: Psychosocial and Biochemical Methods. Totowa, NJ: Humana Press; 1992 p. 41–72.

[35] Rumpf H-J, Hapke U, Meyer C, John U. Screening for alcohol use disorders and at-risk drinking in the general population: psychometric performance of three questionnaires. Alcohol Alcohol. 2002;37(3):261–8. 10.1093/alcalc/37.3.261 12003915

[36] Reinert DF, Allen JP. The alcohol use disorders identification test: an update of research findings. Alcohol Clin Exp Res. 2007;31:185–99. 10.1111/j.1530-0277.2006.00295.x 17250609

[37] National Institute on Alcohol Abuse and Alcoholism. Rethinking drinking: Alcohol and your health. National Institutes of Health; 2010.

[38] Box GEP, Tidwell PW. Transformation of the independent variables. Technometrics. 1962;4:531–50.

[39] StataCorp. Stata Statistical Software: Release 14. College Station, TX: StataCorp LP; 2015.

[40] Krumpal I. Determinants of social desirability bias in sensitive surveys: a literature review. Qual Quant. 2013;47:2025–47. 10.1007/s11135-011-9640-9

[41] Bundesamt Statistisches. Bildungsstand der Bevölkerung—Ergebnisse des Mikrozensus 2016. Wiesbaden: Statistisches Bundesamt, 2018.

[42] Universität Greifswald. Zahlen, Daten, Fakten 2019. https://www.uni-greifswald.de/universitaet/information/zahlen-fakten/zahlen-daten-fakten/.

[43] Statistisches Landesamt Mecklenburg-Vorpommern. Entwicklung der Einwohnerzahl in Greifswald von 1990 bis 2017 2019. https://de.statista.com/statistik/daten/studie/475157/umfrage/entwicklung-der-gesamtbevoelkerung-in-greifswald/.

[44] McNeely J, Strauss SM, Rotrosen J, Ramautar A, Gourevitch MN. Validation of an audio computer-assisted self-interview (ACASI) version of the alcohol, smoking and substance involvement screening test (ASSIST) in primary care patients. Addiction. 2016;111(2):233–44. 10.1111/add.13165 26360315 PMC4899945

